# RGS19 upregulates Nm23-H1/2 metastasis suppressors by transcriptional activation via the cAMP/PKA/CREB pathway

**DOI:** 10.18632/oncotarget.19509

**Published:** 2017-07-22

**Authors:** Yuanjun Li, Jiaxing Song, Yao Tong, Sookja Kim Chung, Yung H. Wong

**Affiliations:** ^1^ Division of Life Sciences and the Biotechnology Research Institute, Hong Kong University of Science and Technology, Hong Kong, China; ^2^ School of Biomedical Sciences, University of Hong Kong, Hong Kong, China; ^3^ State Key Laboratory of Molecular Neuroscience and the Molecular Neuroscience Center, Hong Kong University of Science and Technology, Hong Kong, China; ^4^ Guangdong Provincial Key Laboratory of Brain Science, Disease and Drug Development, HKUST Shenzhen Research Institute, Shenzhen, China

**Keywords:** Nm23, RGS19, transcriptional regulation, PKA

## Abstract

The Nm23 metastasis suppressor family is involved in physiological and pathological processes including tumorigenesis and metastasis. Although the inverse correlation of Nm23 level with tumor metastasis potential has been widely observed, the mechanisms that regulate the expression of Nm23 remain poorly understood. Our previous studies have revealed that Nm23-H1/2 isoforms are upregulated by RGS19, a regulator of G protein signaling (RGS) protein which accelerates the termination of G_i_ signals. Here, we examined the ability of RGS19 to stimulate transcriptional regulation of Nm23 by screening a panel of luciferase reporter genes. Transient and stable overexpression of RGS19 upregulated the Nm23-H1/2 protein levels and activated several transcription factors including CREB, AP-1 and SRE in HEK293 cells. Interestingly, agents that increase the intracellular cAMP level and the phosphorylation of CREB (e.g., adrenergic receptor agonist, forskolin, and cAMP analogues) upregulated the expression of Nm23-H1/2 in HEK293 cells and several cancer cell lines including A549, HeLa, MDA-MB-231, and MDA-MB-435s cells. Conversely, inhibition of protein kinase A (PKA) by H-89 suppressed the phosphorylation of CREB and reduced the expression of Nm23-H1/2. Furthermore, activation of PKA attenuated cancer cell migration in wound healing and transwell assays. Collectively, these results revealed a PKA-dependent mechanism for controlling Nm23-H1/2 expression.

## INTRODUCTION

Metastasis suppressor genes, which exhibit a reduced expression in metastatic tumor cells, are defined by their ability to inhibit metastatic dissemination without impairing primary tumor growth [1]. The prototypical metastasis suppressor is Nm23 which has the ability to down-regulate metastasis formation independently of primary tumor size [2]. The ten members of the Nm23 family are involved in diverse physiological and pathological processes, ranging from proliferation, differentiation, development, ciliary functions to metastasis. Among them, the two most abundantly expressed isoforms are Nm23-H1 and Nm23-H2 [3]. In human cohort studies, low Nm23-H1 expression is correlated with high metastatic potential in a subset of melanoma, liver, colon, and breast cancers [4]. Its role as a metastasis suppressor is strongly indicated by loss of expression at the early stages of the metastatic cascade [5], and the ability to reduce metastasis in a variety of cancer cell models [2, 6].

Given the strong inverse correlation between cancer metastasis and the expression level of Nm23-H1/2, an ability to manipulate the expression of Nm23 proteins may have profound impact on curbing metastasis. However, little is known with regard to the regulatory mechanisms that control the expression of Nm23. Small molecules such as all-trans retinoic acid, medroxyprogesterone acetate (MPA), and a variant of vitamin D have been reported to upregulate the expression of Nm23 in various cell types, while thyroid hormone has been shown to downregulate Nm23 [7–10]. Furthermore, inhibition of DNA methylation apparently participates in the elevation of Nm23-H1 expression [11]. Analysis of the promoter regions of *NME1* and *NME2* (encoding for Nm23-H1 and H2, respectively) have revealed binding sites for several known transcription factors including AP-1, Oct-2 (*NME1*) and PuF (*NME2*) [12, 13]. Using molecular analysis of the Nm23-H1 promoter with sequential deletions, a 248 bp fragment of the promoter containing a MAF/Ets site and a CTF/NF1 half site was implicated in elevating Nm23-H1 expression [11]. As for Nm23-H2, most of the studies on its transcriptional regulation were focused on the Myc binding sites in its promoter region [14]. More recently, we have demonstrated that Nm23-H1/2 can be upregulated by RGS19, a member of the regulators of G protein signaling (RGS proteins), although no clear mechanism was immediately discernable [15].

The ability of RGS19 to upregulate Nm23-H1/2 is rather intriguing. G protein-coupled receptors (GPCRs) have long been recognized as potential oncogenes and tumor suppressors by regulating G protein signaling networks that modulate cell proliferation, and drugs targeting GPCRs have been utilized in cancer therapy [16]. Rapid termination of G protein signals by RGS proteins can potentially modulate growth signals and hence tumorigenesis. Multiple RGS proteins are known to be differentially expressed between normal and cancerous tissues in a variety of cancers, including ovarian cancer and melanoma [17]. Interestingly, RGS19 appears to suppress tumorigenesis via upregulating Nm23-H1/2 [15, 18]. The enhanced expression of Nm23-H1/2 leads to the phosphorylation and translocation of a scaffolding protein, kinase suppressor of Ras (KSR), which is required for efficient propagation of growth signals along the Ras/Raf/MEK/ERK axis [15]. Since RGS proteins are not generally known to modulate the expression of other proteins, the upregulation of Nm23-H1/2 by RGS19 provides an invaluable opportunity to examine whether RGS proteins can indirectly regulate transcription. In this study we present evidence that activation of the cAMP/PKA/CREB pathway can lead to increased expression of Nm23-H1/2, which may provide a new approach to control the process of cancer metastasis.

## RESULTS

### RGS19 activates several transcription factors with putative binding sites on the promoter regions of *NME1/2*

**Figure 1:**
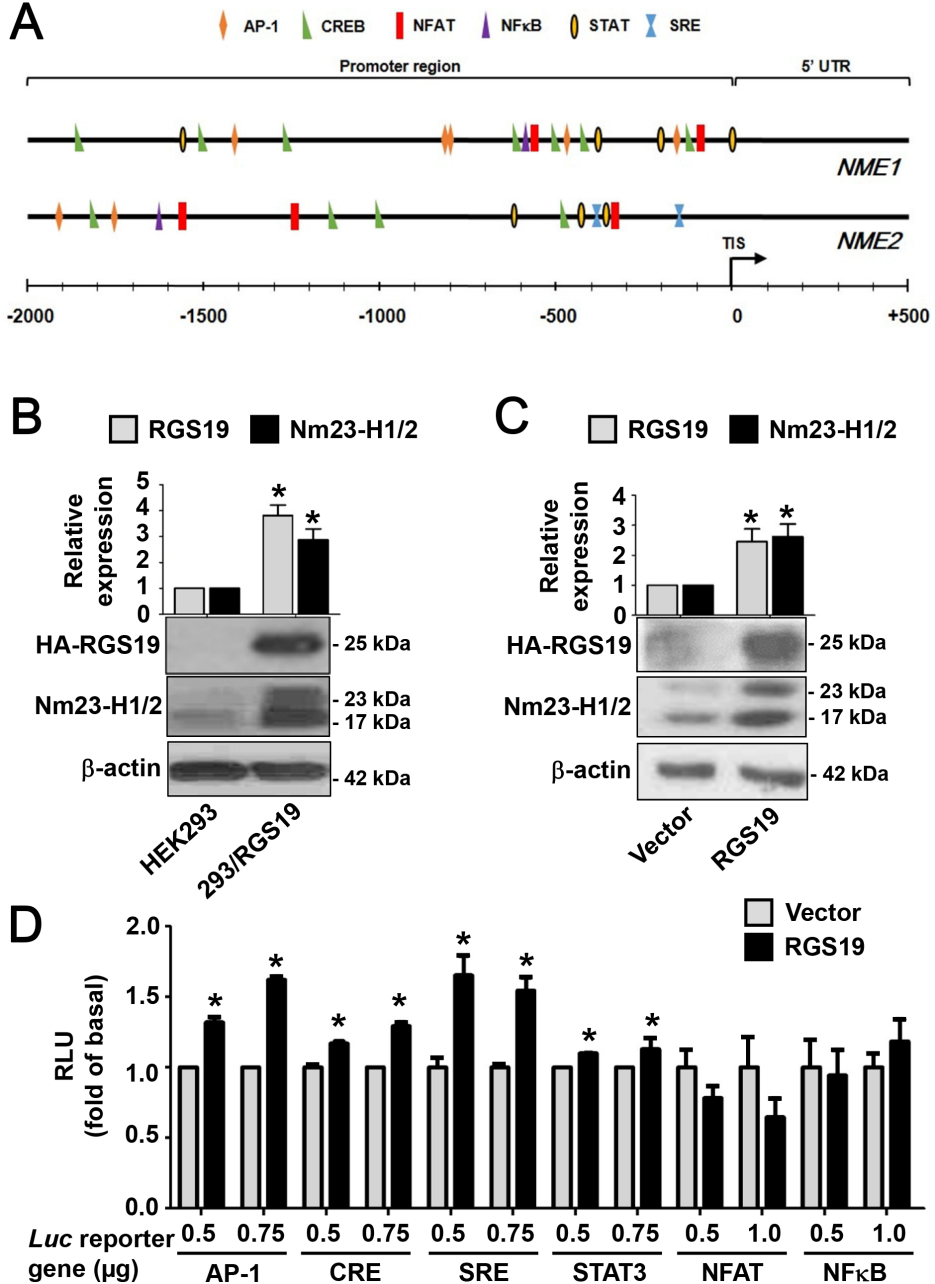
RGS19 upregulates the protein level of Nm23-H1/2 and activates the transcription factors AP-1, CRE, SRE and STAT3 **(A)** Locations of G protein signaling-associated transcription factor binding sites on *NME1/2* promoter regions were predicted by the MatInspector program. TIS, translational initiation site; 5’-UTR, 5’-untranslated region. **(B)** Lysates of HEK293 and 293/RGS19 cells were subjected to Western blot for detecting Nm23-H1/2 and HA-RGS19 with anti-Nm23H1/2 and anti-HA antibodies, respectively. Representative immunoblots are shown. β-actin was used as an internal control; quantification of mean band intensities by Image J is shown on top with the values normalized against the control (parental cell). *RGS19 and Nm23-H1/2 expressions in 293/RGS19 cells are significantly higher than those of HEK293 cells (P < 0.05). **(C)** HeLa cells were transiently transfected with cDNAs encoding HA-RGS19 or vector (pcDNA3.0) and lysates were assayed as in *B*. **(D)** Activation of transcriptional factors by transient expression of RGS19 in HEK293 cells. HEK293 cells were transiently co-transfected with either HA-RGS19 or vector in combination with different luciferase reporter genes (at 0.5, 0.75, or 1.0 μg) as indicated. Transfected cells were cultured for 24 h and lysates were subjected to luciferase assay. Data shown are relative values of luciferase normalized to the respective vector controls. Each bar represents the average of triplicate determinations ± S.E. RLU, relative luciferase unit. *Significantly higher than the vector control (P < 0.05).

Since we have previously shown that the transcript levels of Nm23-H1/2 variants are significantly elevated in HEK293 cells stably expressing RGS19 (henceforth referred to as 293/RGS19) [15], it seems likely that RGS19 can stimulate the transcription of Nm23-H1/2. The promoter region of *NME1* is around 2.1 kb and that of *NME2* is less than 4 kb [11, 14, 19]. Early studies have identified a number of binding sites for transcription factors in the promoter regions of *NME1* and *NME2* [11-13, 20]. Using the matrix-based MatInspector analysis, a number of transcription factor binding sites were found in the promoter regions of *NME1* and *NME2*. In order to focus on the putative transcriptional activity specifically driven by RGS19, several transcription factors were selected on the basis of their potential regulation by G protein signals and possible interaction with Nm23-H1/2 (Table 1). As an RGS protein, RGS19 serves as a GAP (GTPase-activating protein) for Gα_i/o_, Gα_q_, Gα_t_, and Gα_z_ with differential inhibitory potentials [21]. Transcription factors such as NFκB [22, 23], NFAT [24], and STAT3 [25] are known to be regulated by G_i_-coupled receptors. CREB, AP-1 and SRF were also selected in view of their linkage to Gα_s_ and Gα_q_ signals [26]. All of the selected transcription factors are predicted to bind to the promoter regions of *NME1/2* at multiple sites (Table 1) and their relative locations with respect to the translation initiation site (TIS) are depicted in Figure 1A.

**Table 1:** Transcription factors associated with G protein signaling that are predicted to be present in the promoter regions of *NME1* and *NME2*

TFs	Relevant G protein subunits	No. of predicted TF binding sites^*^		Relation to Nm23-H1/2 expression	References
		*NME1*	*NME2*		
AP-1	G_q_, G_12_	5	2	Presence of multiple AP-1 sites on *NME1* promoter	Okada et al., 1996 [12]; Chen et al., 1994 [13]; de la Rosa et al., 1996 [20]
CREB	G_s_	7	4		
NFAT	G_q_, G_βγ_	2	3		
NFκB	G_i/o_, G_q_	1	1	CARMA3 represses *NME2* via NFκB	Chang et al., 2015 [40]
SRE	G_12_, G_q_	0	2		
STAT	G_s_, G_q_, G_βγ_	4	3	Negative feedback between STAT3 and Nm23-H1	Gong et al., 2013 [36]

* Numbers of binding sites are predicted by MatInspector.

Prior to testing the activity of transcription factors by means of reporter gene assays, we first confirmed that 293/RGS19 cells have elevated levels of Nm23-H1/2. As shown in Figure 1B, the expression of Nm23-H1 (23 kDa) and Nm23-H2 (17 kDa) in 293/RGS19 cells was indeed significantly higher (∼3-fold) than those of HEK293 cells. Similarly, transient expression of RGS19 in HeLa cells also led to a higher expression level of Nm23-H1/2 as compared to that of the vector control (Figure 1C). These results confirmed our earlier findings [15] and indicated that upregulation of Nm23-H1/2 by RGS19 may occur in different cell types. To test the potential involvement of the selected transcription factors, we transiently expressed different luciferase reporter genes with or without RGS19 in HEK293 cells. Increasing amounts of luciferase reporter genes were used in the transfections to ensure that the reporter constructs would not become limiting. It was also confirmed that different DNA amounts of *luciferase (luc)* reporter gene did not affect the expression level of RGS19 (Supplementary Figure 1). The luciferase activities driven by AP-1, CRE, SRE or STAT3 were significantly stimulated in RGS19 co-expressing cells as compared to that of the vector controls (Figure 1D); the activities of pCRE-*luc* and pSTAT3-*luc* were weaker than those of the other two reporters. In contrast, neither NFκB nor NFAT driven luciferase activity was stimulated in RGS19 co-expressing cells.

### AP-1, CRE and SRE transcription factors are activated in 293/RGS19 cells

**Figure 2:**
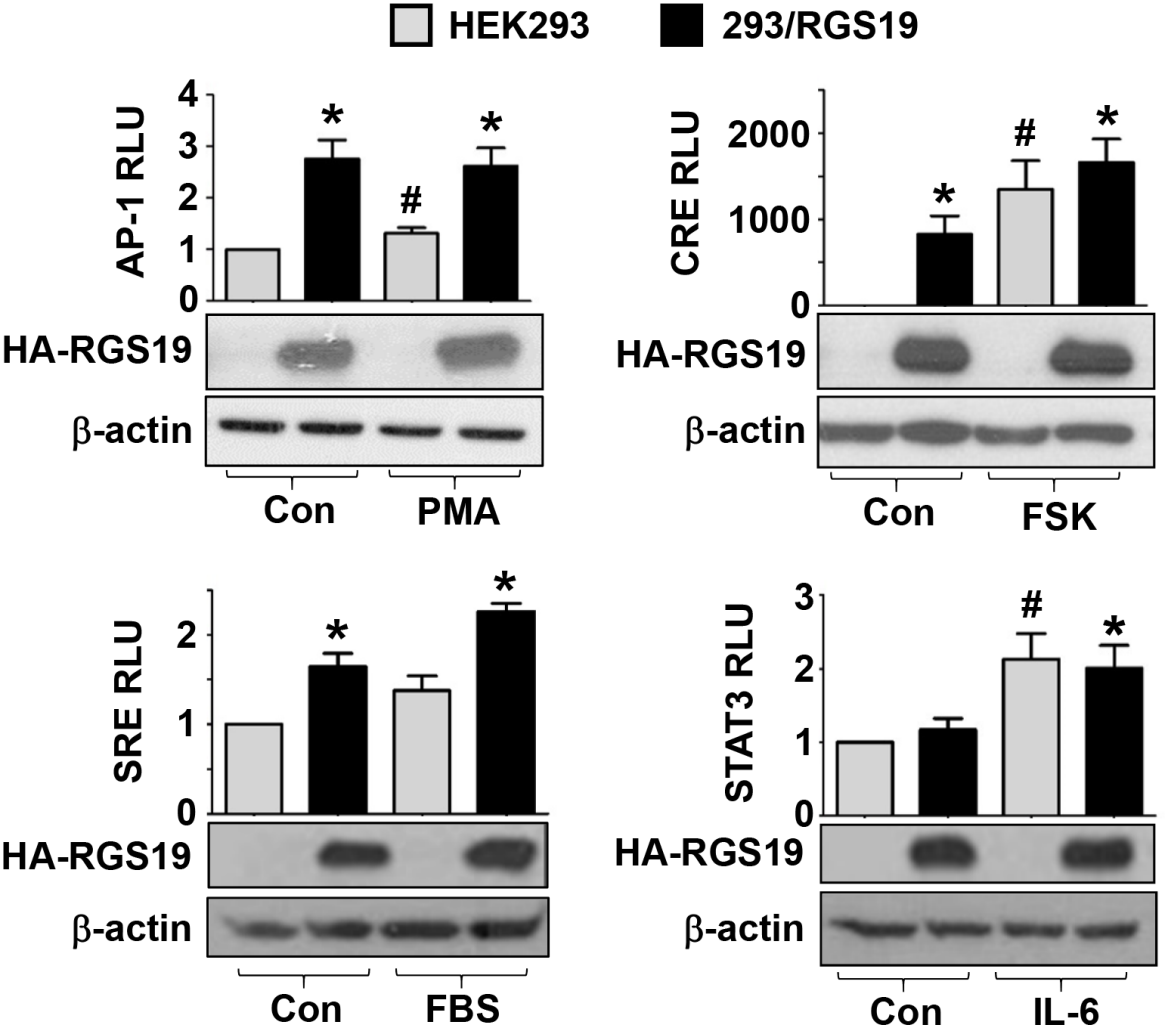
Stable overexpression of RGS19 activates the transcription factors AP-1, CRE, SRE but not STAT3 HEK293 and 293/RGS19 cells were transfected with the indicated luciferase reporter genes. Transfectants were treated with specific agonists targeting different transcription factors: PMA (10 nM, 6 h) for AP-1, forskolin (50 μM, 2 h) for CRE, serum (20%, 6 h) for SRE, and IL-6 (10 ng/ml, 6 h) for STAT3. For PMA and forskolin, 0.1% DMSO was used as the control (Con). Serum-free medium and ddH_2_O solvent were used as the control (Con) for serum and IL-6 treatment, respectively. Lysates were then subjected to luciferase assays. Data shown are relative luciferase units (RLU) normalized to the respective controls (HEK293 with no drug treatment); variations within control groups are <10%. Expression of HA-RGS19 was assessed by Western blots. *Significantly higher than the parental HEK293; #Significantly higher than vehicle treatment (P < 0.05). Each bar represents the mean ± S.E from three independent sets.

The preceding experiments suggest that AP-1, CRE, SRE, and STAT3 transcription factors might be stimulated by RGS19. If this is indeed true, then 293/RGS19 cells should exhibit elevated luciferase activities when the corresponding reporter genes are introduced. Hence, the different reporter genes were transiently transfected into HEK293 or 293/RGS19 cells and the basal as well as stimulated luciferase activities were determined. Basal (control) AP-1, CRE, and SRE driven luciferase activities were significantly higher in 293/RGS19 cells than the parental HEK293 cells (Figure 2). Although basal AP-1 and SRE activities in 293/RGS19 cells were only elevated by less than threefold, basal CRE activity was robustly increased by several hundredfold (Figure 2). Known activators of these reporter genes also exhibited similar levels of stimulation, with phorbol 12-myristate 13-acetate (PMA) and FBS weakly stimulated AP-1 and SRE reporters in HEK293 cells, while forskolin strongly activated the CRE reporter (Figure 2). On the other hand, the STAT3-dependent luciferase activity in 293/RGS19 cells was no different than that of HEK293 cells (Figure 2). Nevertheless, STAT3 luciferase activity was effectively stimulated by IL-6, indicating that the reporter gene was fully functional in the transfectants. In each case, the expression of RGS19 in 293/RGS19 cells was unaffected by the drug treatments (Figure 2). Intriguingly, the magnitude of CREB activation in the 293/RGS19 stable line was much higher than that of transient overexpression of RGS19 (Figures 1D and 2). One possible explanation is that transient transfections do not allow sufficient time to accumulate activated CREB, whereas the relatively long selection time required for establishing stable 293/RGS19 cells would have ensured the buildup of phosphorylated CREB for efficient stimulation of the pCRE-*luc* reporter gene.

### Activation of PKA pathway upregulates the expression of Nm23-H1/2

**Figure 3:**
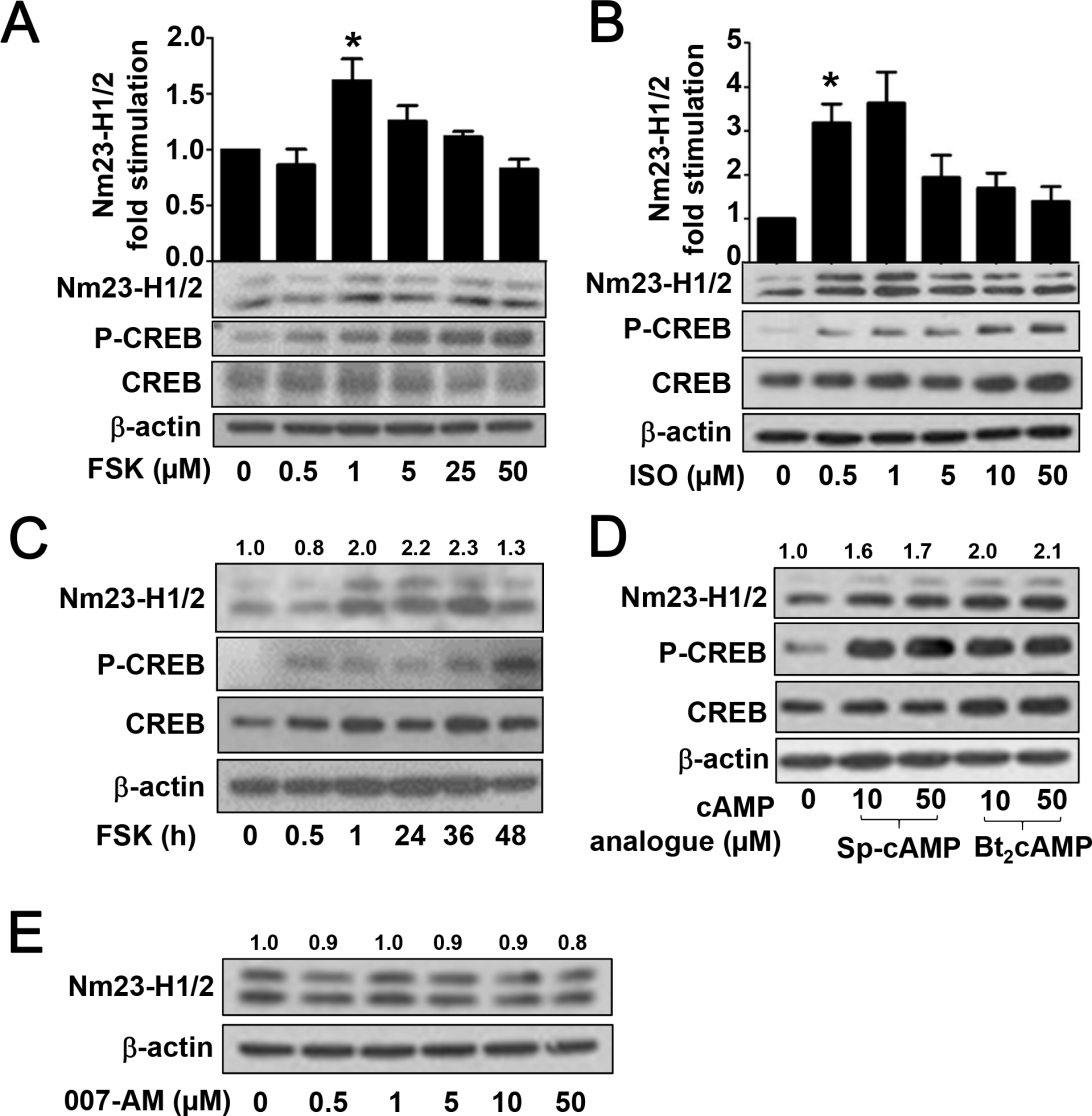
Activation of PKA pathway upregulates the protein level of Nm23-H1/2 **(A)** HEK293 cells were serum-starved overnight, followed by vehicle or forskolin (0.5, 1, 5, 25, or 50 μM) treatment for 24 h. Cell lysates were immunoblotted for detecting Nm23-H1/2, CREB and phosphorylated CREB. **(B)** HEK293 cells were treated as in *A* except forskolin was replaced by isoproterenol (ISO; 0.5, 1, 5, 10, or 50 μM) for 24 h. **(C)** HEK293 cells were serum-starved overnight, followed by 1 μM forskolin treatment for different periods of time. **(D)** HEK293 cells were treated as in *A* except forskolin was replaced by Sp-cAMP (10, 50 μM) or Bt_2_cAMP (10, 50 μM). **(E)** Same as in *A* except forskolin was replaced by 007-AM (0.5, 1, 5, 10, or 50 μM). Numerical values shown above the immunoreactive bands represent relative intensities of Nm23-H1/2 as a ratio of the basal level (vehicle-treated set as 1.0); variations within control groups are <10%. *Forskolin and ISO significantly upregulated the protein level of Nm23-H1/2 as compared to vehicle treatment (P < 0.05). Data shown represents the mean ± S.E from three independent sets; two other sets yielded similar results.

Since CRE sites are predicted to be present in the promoter regions of *NME1* and *NME2* (Figure 1A) and basal CRE activity was significantly increased in RGS19 transient transfectants (Figure 1D) and 293/RGS19 cells (Figure 2), it seems plausible that Nm23-H1/2 expression can be regulated by the cAMP/PKA pathway which lies upstream of the CREB transcription factor. To test this possibility, we employed several approaches to manipulate the production of intracellular cAMP and the function of its canonical target. We first asked if forskolin, a potent direct activator of adenylyl cyclase (AC), can upregulate the protein level of Nm23-H1/2 in HEK293 cells. HEK293 cells were treated with increasing concentrations of forskolin (0.5 to 50 μM) for 24 h and then lysed for the immunodetection of Nm23-H1/2 and CREB phosphorylation by specific antisera. Expression of Nm23-H1/2 was significantly increased in HEK293 cells treated with 1 μM forskolin for 24 h (Figure 3A). Although increased expression of Nm23-H1/2 was also detected in some experiments at 5 μM forskolin, higher concentrations failed to elevate Nm23-H1/2 expression. Forskolin also increased the level of phosphorylated CREB at Ser^133^ without affecting the total CREB (Figure 3A).

Similarly, isoproterenol (ISO), an agonist of β_2_-adrenergic receptor, induced the upregulation of Nm23-H1/2 at relatively low concentrations of 0.5 μM and increased CREB phosphorylation without altering the total amount of CREB (Figure 3B). Although ISO treatment at higher concentrations (5, 10 or 50 μM) remained effective in activating CREB, induction of Nm23-H1/2 became weaker (Figure 3B). In addition, a time-course experiment showed that the upregulation of Nm23-H1/2 protein in HEK293 cells became detectable after 1 h of forskolin treatment, which remained elevated up to 36 h before trailing off by 48 h (Figure 3C). CREB Ser^133^ phosphorylation was detected as early as 30 min of forskolin treatment, indicating that activation of CREB preceded upregulation of Nm23-H1/2 (Figure 3C). CREB phosphorylation remained elevated up to 48 h, consistent with the Nm23-H1/2 upregulation. In addition, cAMP analogues were utilized to investigate their potential stimulatory effect on the expression of Nm23-H1/2. Dibutyryl cAMP (Bt_2_cAMP), and Sp-adenosine 3’,5’-cyclic monophosphorothioate (Sp-cAMP) are potent, phosphodiesterase-resistant, membrane-permeable activators of PKA that are often used to mimic the effects of cAMP. Similar to forskolin and ISO, applications (10 or 50 μM for 24 h) of either cAMP analogues increased the protein expression level of Nm23-H1/2 in HEK293 cells (Figure 3D). CREB Ser^133^ phosphorylation was also elevated by the cAMP analogues.

Besides the classic PKA/CREB cascade, cAMP also targets exchange proteins activated by cAMP (EPAC). To investigate possible involvement of EPAC in regulating cAMP-induced Nm23-H1/2 expression, HEK293 cells were treated with an EPAC-specific activator, 007-AM (8-(4-Chlorophenylthio)-2'-O- methyladenosine-3',5'-cyclic monophosphate acetoxymethyl ester) [27]. As compared to the vehicle control, the 007-AM treatment had no effect on inducing the expression of Nm23-H1/2 in concentrations ranging from 0.5 to 50 μM (Figure 3E), indicating that EPAC proteins may not play a role in regulating Nm23 expression. Collectively, these results suggest that an increase in intracellular cAMP level can induce the expression of Nm23-H1/2, presumably via the PKA/CREB pathway.

### Forskolin induces the expression of endogenous Nm23-H1/2 in cancer cells

**Figure 4:**
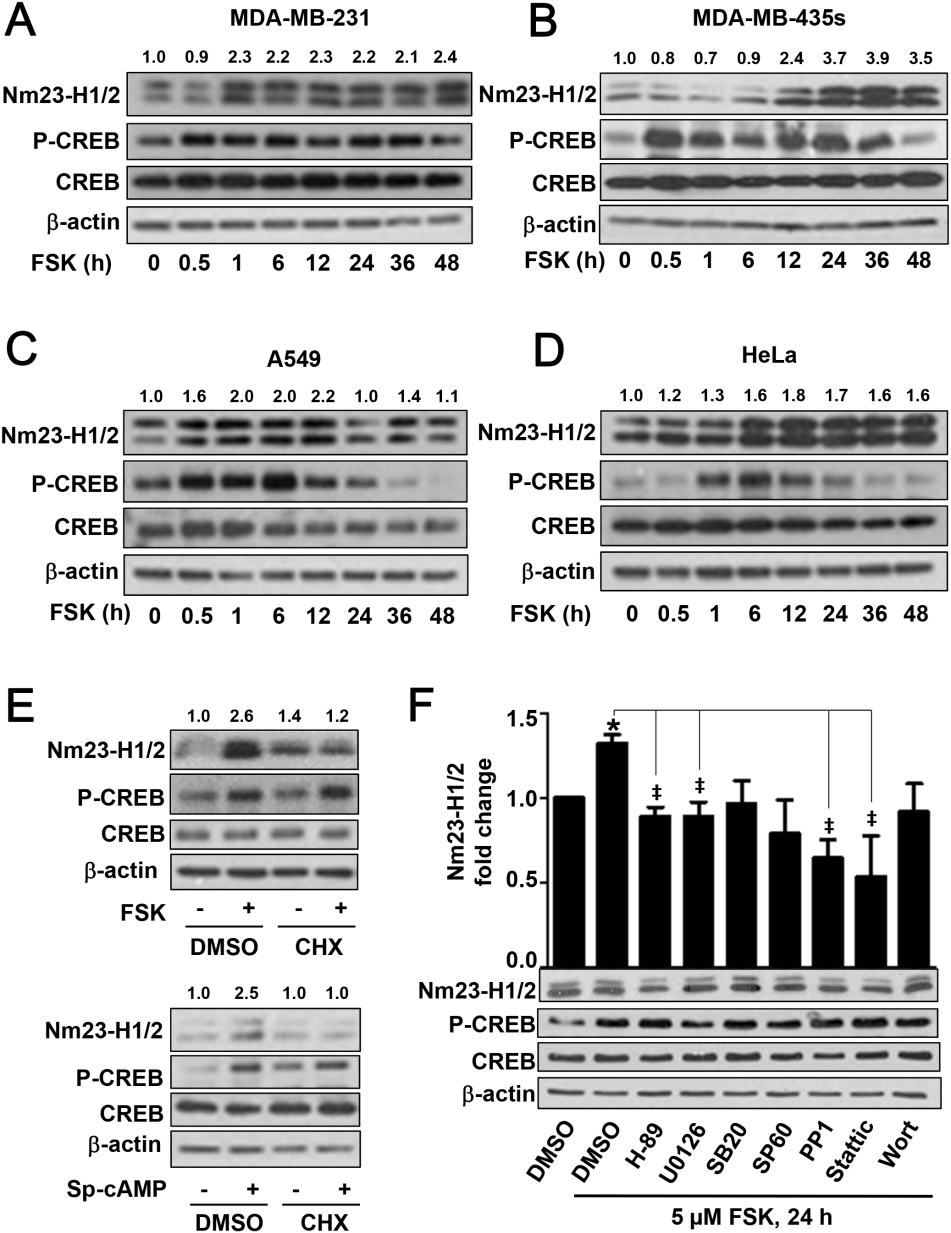
Time-dependent upregulation of Nm23-H1/2 protein expression in various cell lines MDA-MB-231 **(A)**, MDA-MB-435s **(B)**, A549 **(C)** and HeLa **(D)** cells were serum-starved overnight, followed by 5 μM forskolin treatment for different periods of time (0.5, 1, 6, 12, 24, 36, 48 h). Cell lysates were immunoblotted for detecting Nm23-H1/2, CREB and phosphorylated CREB. Numerical values shown above the immunoreactive bands represent relative intensities of Nm23-H1/2 as a ratio of the basal level at time 0 (set as 1.0). **(E)** HEK293 cells were pre-treated with 50 μM cycloheximide (CHX) in serum-free medium for 4 h and then treated with or without 5 μM forskolin or 10 μM Sp-cAMP overnight. **(F)** HEK293 cells were pre-treated with inhibitors specific for different signaling intermediates: 10 μM H-89 (PKA), 10 μM U0126 (ERK), 10 μM SB203580 (p38), 10 μM SP600125 (JNK), 10 μM PP1 (c-Src), 5 μM Stattic (STAT3), and 100 nM wortmannin (PI3K) for 4 h, followed with 5 μM forskolin treatment overnight. Cell lysates were subjected to Western blotting. *Forskolin significantly upregulated the protein expression of Nm23-H1/2 as compared to the vehicle control (P < 0.05); variations within control groups are <5%; ‡Significantly inhibited as compared to forskolin alone (P < 0.05).

Since induction of Nm23-H1/2 in tumor cells may provide a means to suppress metastasis, we examined the ability of forskolin to elevate the expression of Nm23-H1/2 in several cancer cell lines. They include the human metastatic breast cancer MDA-MB-231 and MDA-MB-435s cells, lung adenocarcinoma A549 cells, and cervical cancer HeLa cells. Endogenous expression of Nm23-H1/2 in each of these cell lines was confirmed by immunodetection (Supplementary Figure 2A). Time course experiments revealed that 5 μM forskolin treatment for 1 h or more stimulated the expression of Nm23-H1/2 in MDA-MB-231 cells (Figure 4A). Forskolin-induced CREB Ser^133^ phosphorylation in these cells was detected at 30 min and beyond (Figure 4A). Likewise, forskolin stimulated the expression of Nm23H1/2 in MDA-MB-435s, A549 and HeLa cells, with the appearance of CREB Ser^133^ phosphorylation preceding the upregulation of Nm23-H1/2 (Figures 4B-4D). In the time-course experiments, the peak of Nm23-H1/2 protein expression nearly always appeared after the phosphorylation of CREB, which suggests that Nm23 protein may begin to be expressed sometime after CREB becomes activated. Amongst the cell lines examined, the magnitude of forskolin-induced Nm23-H1/2 upregulation was weakest in HeLa cells. This marginal increase may be attributed to relatively low expression of endogenous Nm23-H1/2 in HeLa cells (Supplementary Figure 2A) which required longer exposure time for image development. Induction of Nm23 protein expression is probably mediated via transcriptional activation because increased Nm23-H1 transcript level can be detected by RT-PCR (Supplementary Figure 2B). The preceding experiments suggest that upregulation of Nm23-H1/2 is likely to occur via transcriptional activation, and will therefore require protein synthesis. Indeed, in HEK293 cells pretreated with cycloheximide (CHX; 50 μM for 4 h) to block protein synthesis, neither forskolin nor Sp-cAMP was able to elevate Nm23-H1/2 protein levels (Figure 4E). CHX, however, did not affect the ability of forskolin or Sp-cAMP to stimulate PKA as increased CREB Ser^133^ phosphorylation was clearly detected (Figure 4E). These results collectively indicated that activation of cAMP/PKA induced Nm23-H1/2 protein expression in various cell types including non-cancer and metastatic cancer cell lines via transcriptional regulation.

Apart from CREB, other transcription factors such as AP-1, STAT3 and SRE are apparently activated by RGS19. A complex mechanism may be involved in the upregulation of Nm23 by RGS19. To assess the potential involvement of additional intermediates in the forskolin-induced Nm23 expression, various inhibitors targeting different signaling molecules were used to pre-treat HEK293 cells prior to the induction of Nm23 expression by forskolin. H-89, PP1, U0126 and Stattic pre-treatment significantly inhibited the forskolin-induced Nm23 protein expression, suggesting the possible involvement of c-Src, ERK and STAT3 in cAMP-induced upregulation of Nm23, respectively (Figure 4F). In contrast, SB203580, SP600125 and wortmannin were unable to block the forskolin-induced response, which indicated a lack of involvement of p38 MAPK, c-Jun N-terminal kinase, and phosphoinositide 3-kinase.

### Nm23-H1/2 protein expression is suppressed by PKA inhibitor

**Figure 5:**
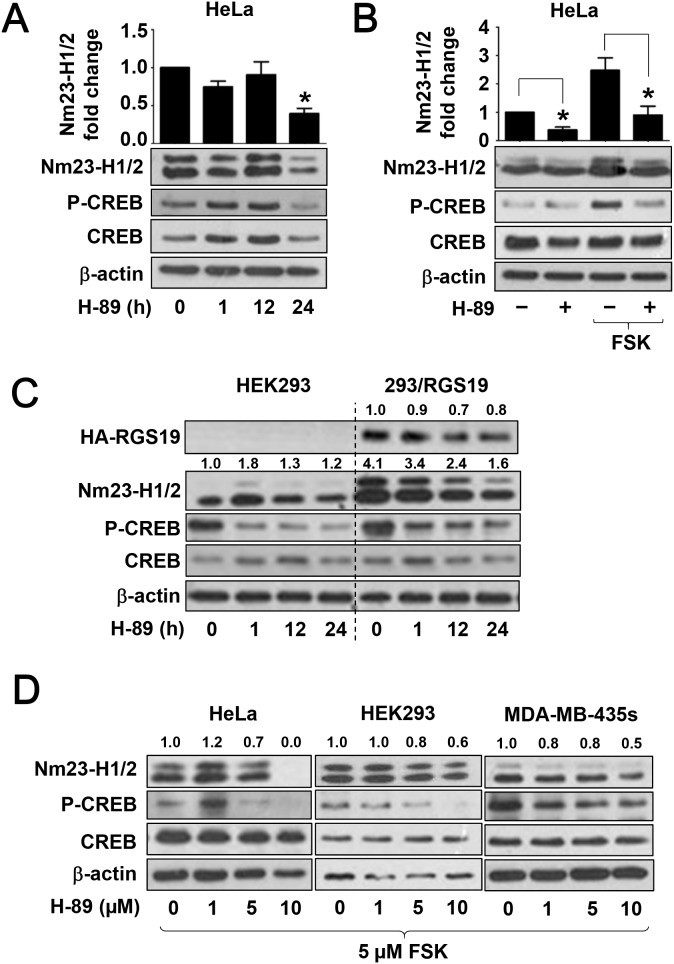
Suppression of Nm23-H1/2 protein level by PKA inhibitor H-89 **(A)** HeLa cells were incubated with 10 μM H-89 or vehicle in serum-free medium for the indicated time. **(B)** HeLa cells were pretreated with 10 μM H-89 or vehicle in serum-free medium overnight, and then incubated with 1 μM forskolin for 24 h. **(C)** HEK293 or 293/RGS19 cells were serum-starved overnight prior to treatment with 10 μM H-89 for the indicated durations. Numerical values shown above the immunoreactive bands represent relative intensities of Nm23-H1/2 as a ratio of the control (HEK293 cells with vehicle treatment). **(D)** HeLa, HEK293, or MDA-MB-435s cells were pretreated with 1, 5, 10 μM H-89 or vehicle in serum-free medium for 4 h, and then incubated with 5 μM forskolin overnight without removal of the H-89. Values shown above the immunoreactive bands represent relative intensities of Nm23-H1/2 as ratio of the control (forskolin alone); variations within control groups are <10%. Cell lysates were immunoblotted for detecting HA-RGS19, Nm23-H1/2, CREB and phosphorylated CREB. Immunoblots shown are representatives of three individual sets of experiments. *H-89 significantly suppressed the expression level of Nm23-H1/2 as compared to the corresponding controls (time zero or vehicle treatment; P < 0.05).

Nextwe used the PKA-specific inhibitor H-89 to test if basal and forskolin-induced Nm23 expression is dependent on PKA activity. We first examined the effect of H-89 on the expression of endogenous Nm23-H1/2 in HeLa cells. As shown in Figure 5A, Nm23-H1/2 levels remained relatively stable up to 12 h of H-89 treatment, but a significant decrease (>60%) was observed at 24 h of treatment. Basal CREB Ser^133^ phosphorylation was also reduced upon H-89 treatment at 24 h. Furthermore, H-89 pre-treatment blocked the forskolin-induced Nm23-H1/2 expression or CREB Ser^133^ phosphorylation (Figure 5B). Because Nm23-H1/2 was upregulated in 293/RGS19 cells, it would be interesting to investigate whether this could be reversed by H-89. Indeed, H-89 treatment significant suppressed the expression of Nm23-H1/2 in 293/RGS19 cells in a time-dependent manner and, likewise, CREB Ser^133^ phosphorylation was reduced by the H-89 (Figure 5C). Unexpectedly, the level of HA-RGS19 in 293/RGS19 cells was also attenuated upon H-89 treatment in a time-dependent manner, with a reduction of 50% by 12 h (Figure 5C). The ability of H-89 to inhibit forskolin-induced Nm23-H1/2 upregulation occurred in a concentration-dependent manner in both HeLa and HEK293 cells (Figure 5D). Moreover, H-89 pretreatment similarly inhibited the forskolin response in MDA-MB-435s metastatic breast cancer cells (Figure 5D). Taken together, these results suggest that PKA signaling plays an important role in modulating the expression level of Nm23-H1/2.

### cAMP/PKA activation suppresses metastatic cancer cell migration

**Figure 6:**
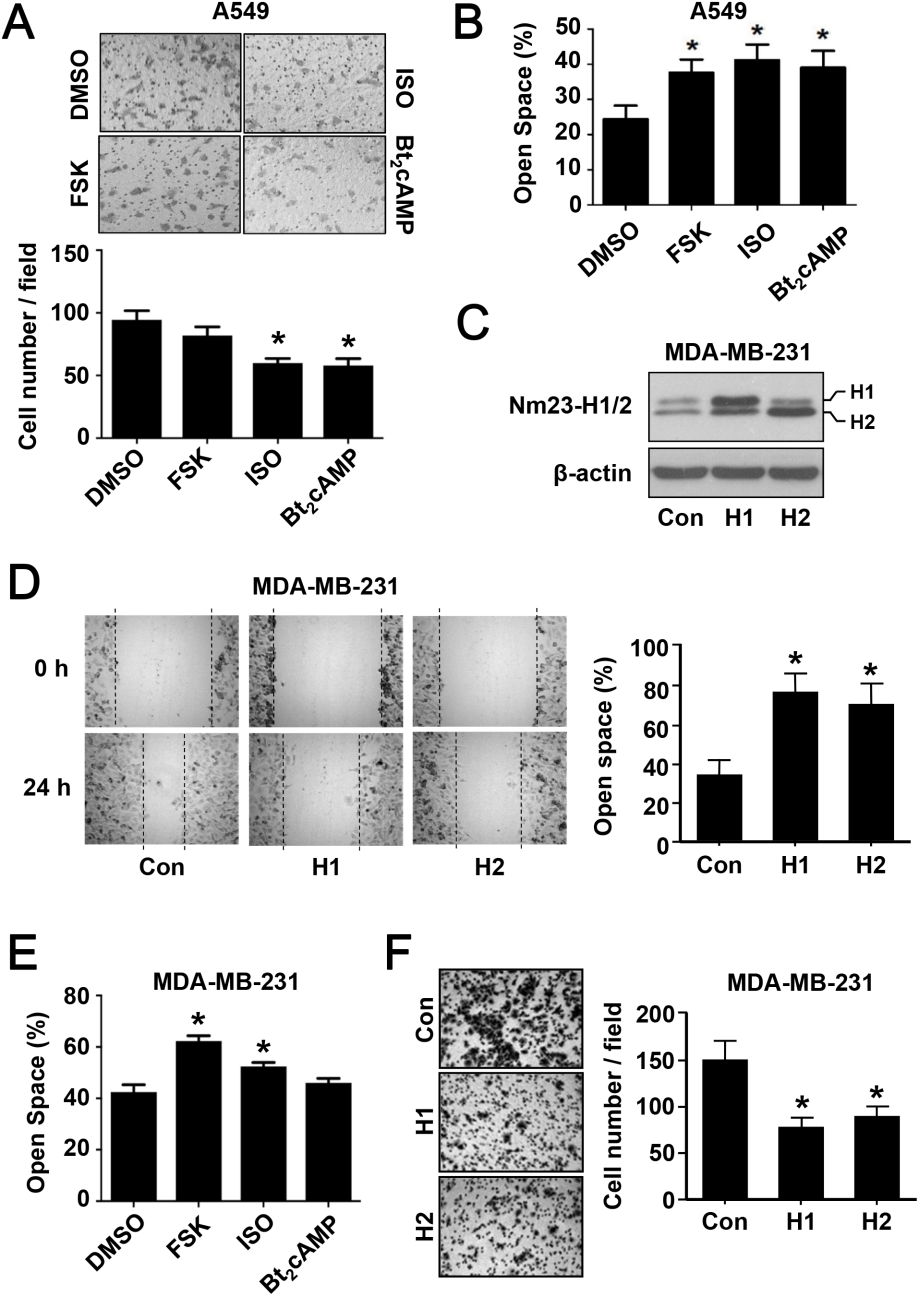
The effect of PKA activation on cancer cell migration **(A)** Metastatic lung cancer A549 cells were subjected to the following drug treatment for 24 h in serum-free medium: vehicle (DMSO, 0.5% v/v), forskolin (FSK, 5 μM), isoproterenol (ISO, 1 μM), and Bt_2_cAMP (10 μM). After the treatment, cells were reseeded into the inserts of transwell plates and allowed to migrate for 24 h prior to fixation and staining. Images were obtained with an Olympus 1X-HOS imaging system at 100× magnification and representative photos of each condition are shown. Quantification of the images is shown below the micrographs. **(B)** Confluent monolayer of A549 cells was scratched with a yellow tip and washed with PBS. Cells were allowed to migrate into the open wound for 24 h in the absence or presence of PKA activating agents as in panel *A*. Images were taken immediately after creating the wound and at 24 h. The open space (%) was the ratio of wounded area not covered by migratory cells after migration to that of time zero. **(C)** Metastatic breast cancer MDA-MB-231 cells stably overexpressing Nm23H1 or Nm23H2 were established as described in Materials and Methods. Cell lysates were analyzed by Western blotting with an anti-Nm23H1/2 antiserum. **(D)** MDA-MB-231 cells overexpressing Nm23H1 or Nm23H2 were subjected to the wound healing assay. **(E)** Parental MDA-MB-231 cells were treated with PKA activators and assayed as in panel *B*. **(F)** MDA-MB-231 cells overexpressing Nm23H1 or Nm23H2 were subjected to transwell migration assay as in panel *A*. *Significantly different to that of the corresponding vehicle or control group (P < 0.05).

As a metastasis suppressor, Nm23 is capable of inhibiting cell migration [28]. We thus asked whether the upregulation of Nm23-H1/2 by cAMP/PKA activation would affect the migration of cancer cells. Activation of the cAMP/PKA pathway by ISO or Bt_2_cAMP significantly inhibited the migration of A549 cells in the transwell migration assay, whereas forskolin elicited a weak inhibitory effect (Figure 6A). Also, wound healing assay showed that activation of the cAMP/PKA pathway significantly suppressed the migration of A549 cells; forskolin, ISO and Bt_2_cAMP treatment significantly increased the open space as compared to the control group with DMSO treatment (Figure 6B). Since the loss of Nm23H1/2 will invariably enhance cell migration [28], a knockdown approach would not reveal the involvement of Nm23H1/2 downstream of the cAMP/PKA/CREB pathway. To test whether elevated levels of Nm23H1/2 can indeed suppress the mobility of cancer cells, MDA-MB-231 cells stably expressing Nm23H1 or H2 (MDA/H1 or MDA/H2) were established. Expression of Nm23H1/2 was confirmed by Western blot analysis using an anti-Nm23 antiserum (Figure 6C). In the wound healing assay, migration of parental MDA-MB-231 cells gradually reduced the open space to less than 40% by 24 h, whereas the open space of MDA/H1 and MDA/H2 remained at 70% or more (Figure 6D). Parental MDA-MB-231 cells treated with forskolin or ISO also left a broader open space as compared to the DMSO control group (Figure 6E). In the transwell migration assay, MDA/H1 and MDA/H2 cells exhibited reduced mobility (Figure 6F) that resembled the impairment induced by ISO and Bt_2_cAMP treatment (Figure 6A). Taken together, these results illustrate that the activation of cAMP/PKA suppresses the behavior of cancer cell migration.

## DISCUSSION

Numerous studies ranging from knockout mice experiments to clinical cancer cohort trials have clearly demonstrated the multi-functional and metastasis suppressive effects of Nm23 [29], thus paving the way to develop new anti-metastasis therapies by manipulating the expression level of Nm23 [10]. Driven by the recent observation that RGS19 can upregulate the expression of Nm23-H1/2 [15], we have further examined the mechanistic basis of this regulation and herein present several lines of evidence that implicate the involvement of the cAMP/PKA/CREB pathway: (1) Transient or stable expression of RGS19 activated the CRE transcription element which is present on the promoter regions of *NME1/2*; (2) Manipulation of cAMP/PKA/CREB signaling accordingly stimulated or suppressed the transcription and expression of Nm23-H1/2; (3) Agonist-induced stimulation of G_s_-coupled receptors resulted in the upregulation of Nm23-H1/2; and (4) Activation of the cAMP/PKA/CREB pathway suppressed cancer cell migration. Moreover, our data further suggests a complex regulatory mechanism which may involve other signaling intermediates such as c-Src and STAT3.

**Figure 7:**
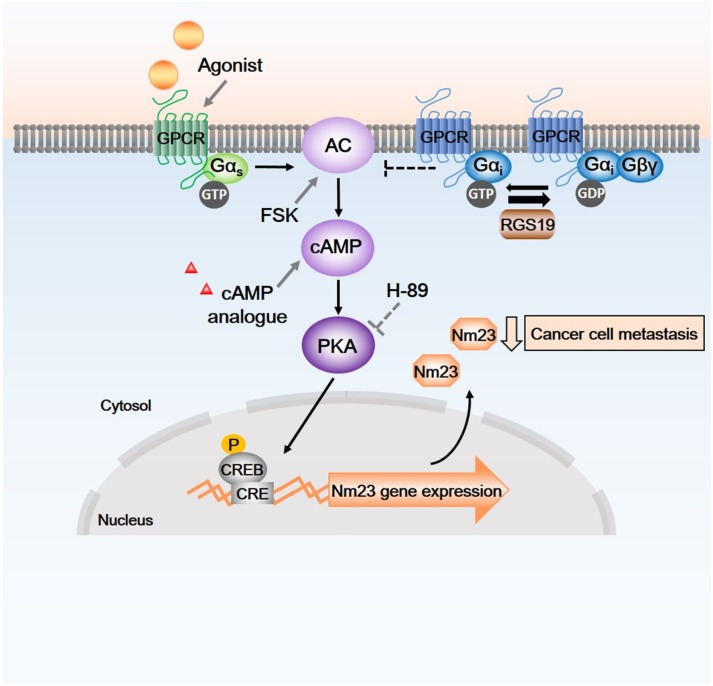
Schematic diagram of possible signaling pathways between RGS19, G proteins, cAMP/PKA cascades and transcription factors As a GAP, RGS19 terminates the action of active GTP-bound Gα_i_, relieves the inhibition of adenylyl cyclase (AC) that produce cAMP. Elevation of intracellular cAMP level and the activation of PKA by agonists of G_s_-coupled receptors, AC activator (forskolin) and cAMP analogues lead to phosphorylation of CREB, thus driving the expression of Nm23-H1/2. Endogenous or forskolin-induced Nm23-H1/2 protein expression can be suppressed by H-89 which inhibits PKA. Arrow with solid line, activation; blunted arrow, inhibition. CRE, cAMP responsive element; CREB, CRE-binding protein; P, phosphate; PKA, cAMP-dependent protein kinase.

In this study, we observed an activation of CREB by RGS19 as demonstrated by both the pCRE-*luc* reporter assay and the phosphorylation of CREB. The ability of RGS19 to stimulate the cAMP/PKA/CREB pathway is rather intriguing and may be mediated indirectly because RGS19 does not seem to significantly affect the formation of cAMP (Supplementary Figure 3). As a GAP for Gα_i/o_ subunits [21], RGS19 can potentially elevate intracellular cAMP by limiting the inhibitory actions of Gα_i_ on AC, thereby allowing Gα_s_ to stimulate the production of cAMP more efficiently (Figure 7). Although such a mechanism is plausible, it would imply that other RGS proteins which also serve as GAPs for Gα_i_ may similarly enhance cAMP accumulation. Expression of RGS17 has indeed been shown to activate the cAMP/PKA/CREB pathway, apparently leading to the growth of pancreatic tumors [30]. However, expression of RGS2, 7, and 8 failed to upregulate Nm23-H1 in HEK293 cells [15], thereby suggesting that the GAP function does not appear to play an important role in the regulation of Nm23 expression. Since suppression of ERK activity is associated with elevated Nm23 levels [31], other Gα_i_-targeting RGS proteins including RGS4, 10, and 20 are probably incapable of upregulating Nm23 because they had little effect on ERK phosphorylation [15]. Taken together, the available information tends to suggest that RGS19 may be unique within the RGS family for its ability to upregulate Nm23-H1/2 in multiple cell types. This postulation, however, would require further confirmation of whether RGS17 possesses the ability to upregulate Nm23.

Irrespective of the mechanistic linkage to RGS19, the present study undoubtedly demonstrated that upregulation of Nm23 can be induced by signals that stimulate the formation of cAMP. This may have significant therapeutic implications because many G_s_-coupled receptors including those for follicle-stimulating hormone, thyroid-stimulating hormone, and luteinizing hormone are intricately associated with tumorigenesis in a variety of cancers. Analogues of luteinizing hormone-releasing hormone represent important options for endocrine treatment of metastatic breast cancer, and their combined use with other drugs such as tamoxifen and MPA have produced beneficial outcomes [32]. MPA has long been known to elevate Nm23-H1 expression and suppress colony formation of MDA-MB-231 and MDA-MB-435s human breast carcinoma cells in the soft agar assay [19]. Our demonstration of a cAMP/PKA/CREB driven upregulation of Nm23 is consistent with the reported ability of MPA to elevate intracellular cAMP and stimulate the PKA pathway in human endometrial stromal cells [33]. Disappointingly, a recent phase II clinical trial of MPA failed to warrant its use in metastatic breast cancer [34]. Since the cAMP/PKA/CREB pathway can exert both growth promoting and inhibiting effects on different types of tumors [35], activation of this pathway is unlikely to be the sole determinant for the upregulation of Nm23.

*In silico* analysis of *NME1/2* promoter regions revealed other important transcription factor binding sites besides CREB, such as STAT3 and NFκB. The potential ability of STAT3 to regulate Nm23 expression warrants further discussion. At least two putative STAT3 binding sites were predicted to be present in the promoter region of *NME1* (Figure 1A), and elevated STAT3 activity was detected upon transient overexpression of RGS19 (Figure 1D), but not in the 293/RGS19 cells (Figure 2). The contradictory effects in transient and stable transfectants may be due to a negative feedback regulatory mechanism between Nm23 and STAT3. IL-6-induced STAT3 activation is significantly enhanced in A549 cells upon siRNA-mediated knockdown of Nm23-H1, an effect which can be prevented by the ectopic expression of shRNA-resistant Nm23-H1 [36]. Thus, stable expression of RGS19 in 293/RGS19 cells might trigger negative feedback regulation on STAT3 because of the sustained elevation of Nm23-H1/2. Since Stattic effectively inhibited the forskolin-induced upregulation of Nm23 in HEK293 cells (Figure 4E), it seems that STAT3 activation is involved in the transcriptional control of Nm23. However, it should also be noted that repression of Nm23-H1 by STAT3 has been demonstrated in human trophoblasts [37]. The dynamic interactions between STAT3 and Nm23 isoforms may provide regulatory mechanisms for fine tuning the expression of Nm23, STAT3, or both. As forskolin-induced upregulation of Nm23 was sensitive to ERK and c-Src inhibition (Figure 4E), additional regulatory controls might be present along the cAMP/PKA/CREB pathway that leads to Nm23 transcription. Precedent of c-Src enhancing ISO-induced cAMP accumulation has indeed been reported in murine fibroblasts [38]. Moreover, both ERK and c-Src are known to stimulate STAT3 activity [39].

Another recent study suggests that Nm23-H2 expression is suppressed by NFκB-dependent induction of miR-182 [40], which is consistent with the lack of NFκB activity in HEK293 cells expressing RGS19 (Figure 1D). Interestingly, a splice variant of Nm23-H1, the NME1L, can negatively regulate NFκB signaling by interacting with IKK [41]. It would appear that the expression of Nm23-H1/2 is tightly controlled by complex regulatory mechanisms wherein the different Nm23 splice variants may play distinct roles. Specific regulation of Nm23 isoforms may be necessary in view of their differential abilities to regulate biochemical and cellular responses [42, 43].

The linkage between RGS19, cAMP/CREB pathway and Nm23-H1/2 expression described herein may provide clues to the *in vivo* effectiveness of cAMP-modifying agents that suppress cancer cell migration [44, 45]. In *in vivo* experimental models, the PDE inhibitor dipyridamole decreases metastasis formation in xenograft animals [46], an effect which may well be mediated via the upregulation of Nm23-H1/2. More interestingly, it has been suggested that PKA participates in the differentiation of tumor-initiating cells by enforcing residence in the epithelial state and reversing the epithelial-to-mesenchymal transition program [47]. In carcinoma cells, entry into a more mesenchymal state is associated with elevated metastatic ability and drug-resistance to conventional chemotherapeutics [48]. Similarly, activation of β_2_-adrenergic receptor by the highly-selective (R, R’)-4’-methoxy-1-naphthylfenoterol [(R, R’)-MNF] inhibits proliferation and motility of melanoma cells [49]. This effect by (R, R’)-MNF was mimicked by ISO, forskolin, and the phosphodiesterase 4 inhibitor Ro 201724 [49], suggesting a crucial role of cAMP/PKA for this phenomenon. However, evidence relating to the cAMP/PKA pathway and cancer metastatic behavior remains somewhat controversial. For instance, β-blockers have been shown to exhibit anti-metastatic effects [50]. The present results demonstrating that cAMP/PKA/CREB activation upregulates metastasis suppressor Nm23-H1/2 in various cancer cell lines may provide a possible mechanism to understand the relationship between the cAMP/PKA/CREB pathway and cancer cell metastasis.

## MATERIALS AND METHODS

### cDNA constructs, cell culture and antibodies

The cDNA of RGS19 was purchased from Missouri S&T cDNA Resource Center (Rolla, MO, USA). The cDNAs of human Nm23H1 and Nm23H2 were kindly provided by Dr. Patricia S. Steeg (National Cancer Institute) and Dr. Tina H. Lee (Carnegie Mellon University), respectively. Luciferase reporter genes pCRE-*luc*, pSRE-*luc* and pSTAT3-*luc* were from Clontech (Palo Alto, CA), and pAP-1-*luc* was kindly provided by Prof. Karl Tsim (Division of Life Science, Hong Kong University of Science and Technology). HEK293, MDA-MB-231, MDA-MB-435s, A549, and HeLa cells were purchased from and authenticated by the American Type Culture Collection (Rockville, MD, USA). Cells were cultured in accordance to ATCC recommendations. Transfections were performed using LipofectAMINE 2000 reagent (Life Technologies, Grand Island, NY) according to the manufacturer’s protocol. For stable transfection in MDA-MB-231 cells, cells were selected by 0.5 mg/mL G418 for 2-3 weeks. Expression of Nm23H1/2 was confirmed by immunoblots. Anti-β-actin (A1978) was from Sigma-Aldrich, anti-HA (11666606001) from Roche, anti-CREB (#9197), anti-phospho-CREB (#9198) and anti-Nm23H1/2 (#3338) were from Cell Signaling Technology.

### *In silico* sequence analysis

The presence of transcription factor binding sites in the putative promoter regions of *NME1/2* was predicted using the MatInspector version 7.1 [51] at *www.genomatix.de*. Sites with scores (core similarity) below 0.75 were not considered [52]. The *NME1* promoter sequence was from GenBank: L35301, L34822; the *NME2* promoter sequence (2000 bp upstream of 5’ flanking sequence) was from Ensemble (NME2 ENSG00000243678). The promoter sequences were confirmed with the UCSC genome browser at *genome.ucsc.edu*.

### Western blot analysis

After transfection or treatment, cells were harvested and lysed in ice-cold lysis buffer containing protease inhibitor cocktail. Cell debris was removed by centrifugation at 14,000 rpm for 3 min at 4°C, and the supernatant was used as total protein lysate. Samples (50 μg protein) were resolved by 12% or 15% SDS-PAGE gel electrophoresis and then transferred to nitrocellulose membrane. Target proteins were detected by their specific primary antibodies and horseradish peroxidase-conjugated secondary antisera. The immunoblots were visualized by chemiluminescence with the ECL kit from Amersham. The level of β-actin was determined as an internal control.

### Luciferase assay

Luciferase assay was performed as described previously [53]. The growth medium of serum-starved transfectants was removed and cells were lysed with lysis buffer provided in the Luciferase Reporter Gene Assay High Sensitivity kit (Roche Applied Science). The luciferase activity was determined by a microplate luminometer LB96V (EG&G Berthold, Germany). Briefly, an injector connected to the luciferin substrate was set to inject 25 μl of substrate into each well. A period of 1 second delaying time followed by a 10 sec measuring time period was assigned after introduction of subtract. Results were collected by WinGlow version 1.24 and expressed as relative luminescent units (RLU).

### Transwell migration assay

Cells were incubated with compounds of interest or vehicle in serum-free medium for overnight and then reseeded at 3 × 10^4^ per well in 100 μl medium without fetal bovine serum (FBS) in the upper chambers of 24-well transwell plates (Corning Inc. New York, USA; 8.0 μm pore size). The lower chambers were loaded with 600 μl growth medium with 10 % FBS. After incubation for 24 h, the inserts were washed with PBS and fixed in 4% paraformaldehyde for 15 min prior to staining with 0.5% crystal violet for another 15 min. Cells at the upper side of the top chamber were swabbed with cotton strips and photos were randomly taken by Olympus 1X-HOS imaging system at 100× magnification.

### Wound healing assay

Wound healing assay was conducted as previously described [54]. Briefly, cancer cells were challenged with compounds of interest or vehicle in serum-free medium for overnight. After washing with PBS, confluent monolayers of cells were scratched with yellow micropipette tips and cultured in growth medium with or without drugs for the indicated periods. Images were captured at the onset of the treatment and at selected time-points by Olympus 1X-HOS imaging system at 100× magnification. The open space (%) was calculated as a ratio of the wounded area after migration to the wounded area before migration. Image J was employed to measure the wounded area.

### Statistical analysis

Data were expressed as the mean ± S.E. of at least three independent sets of experiments. The probability of an observed difference being a coincidence was evaluated by paired Student’s *t* test using GraphPad Prism version 5.01. Differences at values of P < 0.05 were considered significant.

## SUPPLEMENTARY MATERIALS FIGURES


