# Supplementary material for: RGS19 upregulates Nm23-H1/2 metastasis suppressors by transcriptional activation via the cAMP/PKA/CREB pathway
Source: Oncotarget. 2017 Jul 22;8(41):69945–60. doi: 10.18632/oncotarget.19509 (PMC5642529; doi:10.18632/oncotarget.19509)
Supplement: Supplementary file 1 [file oncotarget-08-69945-s001.pdf]

# RGS19 upregulates Nm23-H1/2 metastasis suppressors by transcriptional activation via the cAMP/PKA/CREB pathway

## SUPPLEMENTARY MATERIALS

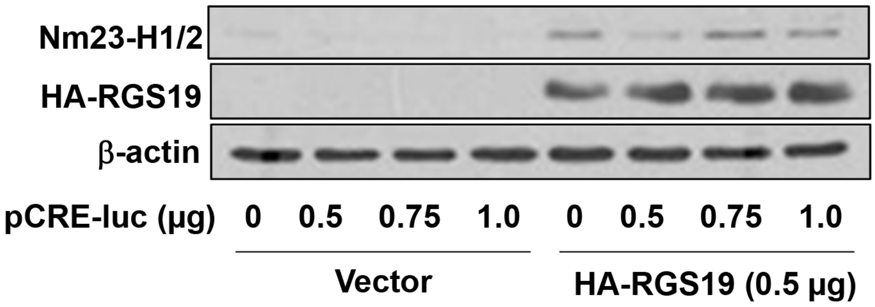


**Supplementary Figure 1: Transient overexpression of RGS19 is unaffected by co-transfection with luciferase reporters.** HEK293 cells were seeded at $3.5 \times 10^5$ cells per well in 6-well plates and transiently co-transfected with either 0.5 μg pcDNA3.0 (Vector) or HA-RGS19 in combination with different amounts (0 to 1.0 μg) of pCRE-*luc* reporter construct. The DNA amount of luciferase reporter gene was balanced with vector plasmid TA-*luc*. Transfectants were cultured for 24 h and cell lysates were subjected to Western blot analysis. Expression of Nm23-H1/2 and HA-RGS19 was confirmed by immunodetection using anti-Nm23 and anti-HA antibodies, respectively. HA-tagged RGS19 expression remained relatively stable among the transfectants with different amounts of CRE-*luc* reporter gene, indicating that the increasing amounts of reporter constructs did not limit the expression of RGS19.

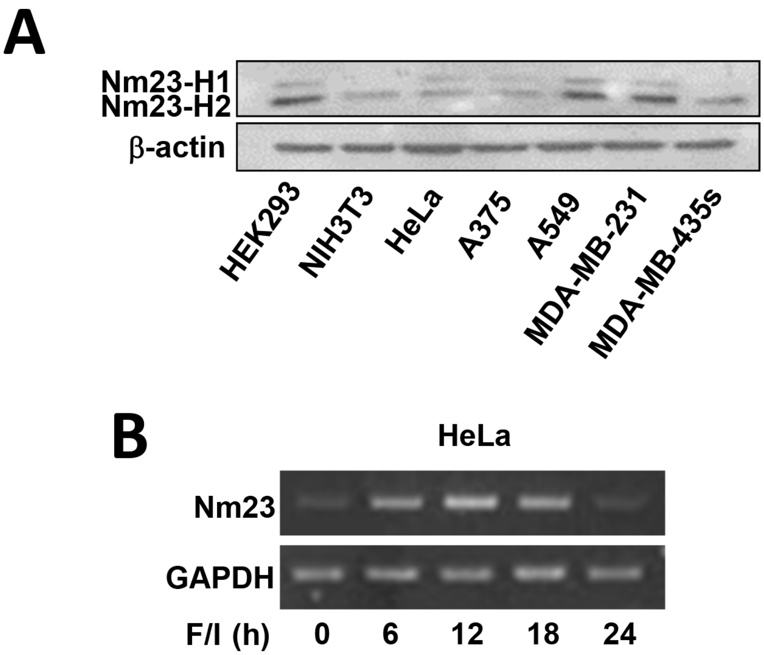


**Supplementary Figure 2: (A)** Endogenous expression levels of Nm23-H1/2 in various mammalian cell lines. Total protein from the indicated cell lines were extracted and equal amounts of lysate proteins were subjected to Western blotting. The endogenous Nm23-H1/2 proteins were detected by an anti-Nm23-H1/2 antiserum. HEK293 cells, A549 and MDA-MB-231 cells show relatively higher expression level of Nm23-H1/2. **(B)** HeLa cells were seeded at $3.0 \times 10^5$ cells per well in 6-well plates one day before and then treated with forskolin (10 μM) in the presence of 3-isobutyl-1-methylxanthine (100 μM) for the indicated durations. Total RNA was extracted with TRIzol reagent (Invitrogen) and the Nm23 transcript level subsequently determined by RT-PCR. SuperScript III First-Strand Kit from Invitrogen (Carlsbad, CA) was used to prepare cDNA samples. Reverse-transcription PCR was performed with KAPA HiFi HotStart ReadyMix. Specific primers for Nm23-H1 (5’-GCG GGG TCT TGC AGC CGA GAT TAT CAA G-3’ forward; 5'-CAC TAG TCC AGT GTG GTG GAA-3' reverse) and GAPDH (5’-TGA TGA CAT CAA GAA GGT GGT GAA G-3’ and 5’-TCC TTG GAG GCC ATG TGG GCC AT-3’) are used. Forskolin induced mRNA level of Nm23-H1/2 from 6 to 18 h.

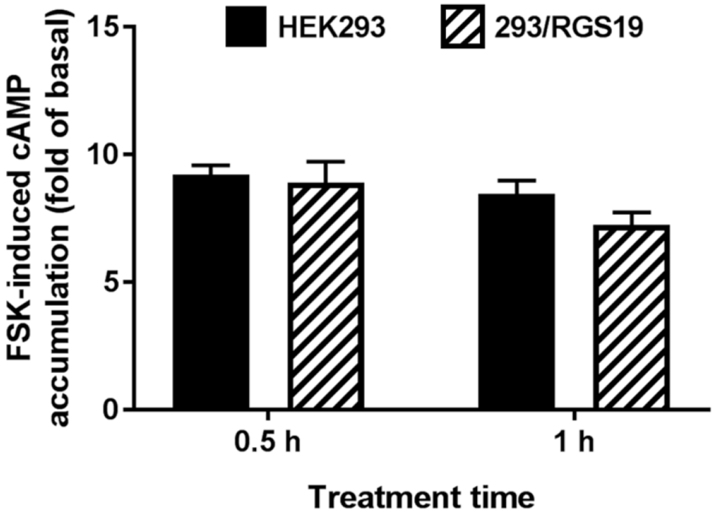


**Supplementary Figure 3: Forskolin-stimulated cAMP accumulation in HEK293 was not affected by the stable expression of RGS19.** HEK293 and 293/RGS19 cells were labeled by [$^3$H]adenine for 24 h. Cells were washed and treated with or without forskolin (FSK; 5 mM) in assay medium containing 1 mM 1-methyl-3-isobutylxanthine for 0.5 h and 1 h. The reaction was terminated by the addition of trichloroacetic acid. [$^3$H]cAMP was isolated by sequential chromatography and estimated by determining the ratios of [$^3$H]cAMP to total [$^3$H]ATP, [$^3$H]ADP and [$^3$H]cAMP pools as previously described (Wong, 1994, *Methods Enzym*. *238*:81-94). Results are expressed as FSK-stimulated cAMP accumulation as compared to the corresponding basal levels. Data shown represents the mean ± S.E. of triplicates from a representative experiment.
